# Salivary cortisol measurements in brachycephalic dog breeds as part of a standardized stress test

**DOI:** 10.3389/fvets.2024.1351225

**Published:** 2024-07-31

**Authors:** Elisa Kähler, Andrea Meyer-Lindenberg, Yury Zablotski, Maike Schroers

**Affiliations:** Veterinary Faculty, Clinic of Small Animal Surgery and Reproduction, Ludwig-Maximilians-Universität München, Munich, Germany

**Keywords:** dog, stress, saliva, cortisol, fitness, test, boas

## Abstract

**Introduction:**

Brachycephalic obstructive airway syndrome (BOAS) is a common condition in brachycephalic dogs, with Pugs (PG) and French Bulldogs (FB) appearing to be particularly typically affected. Objective and easy-to-perform tests are necessary to detect the disease at an early stage and to exclude dogs affected by BOAS from breeding.

**Methods:**

The present study investigated the extent to which vital signs and salivary cortisol concentrations can be used to distinguish between healthy and BOAS-affected dogs in a standardized fitness test. A total of 57 PG, 56 FB and 27 meso- and dolichocephalic dogs were studied as control group (CG). In addition to vital signs, salivary cortisol concentrations were measured before and after the exercise test.

**Results:**

It emerged that non-brachycephalic dogs showed a higher fitness level than brachycephalic dogs. The PG recovered significantly slower than the FB after the exercise test. In unaffected PG, cortisol levels rose significantly after the test and then fell again, in unaffected FB they fell significantly during the test. Unexpectedly, cortisol levels remained constant in BOAS affected dogs of both breeds.

**Discussion:**

A possible explanation could be a disturbance of the pituitary–hypothalamic–adrenal axis, which could be due to the chronic stress of affected animals. This would have to be clarified in further studies. In conclusion, a submaximal fitness test may be a useful method to identify dogs suffering from BOAS as it is imperative to prevent the breeding and reproduction of affected dogs.

## 1 Introduction

The controversial discussion about the health status of brachycephalic dogs represents a very topical issue in veterinary medicine worldwide (1, 2). Recent studies have shown that brachycephalic dogs are predisposed to many diseases compared to non-brachycephalic dogs (3, 4). The popularity of these breeds is associated with “childlike” facial features, including a particularly short nose and large eyes. This led to the selection of anatomical features and abnormalities, such as the relative restriction of airways, narrower nostrils, clogged conches and longer soft palate (5, 6). Such anomalies can often cause chronic respiratory problems in brachycephalic dog breeds, such as the Pug, French Bulldog or English Bulldog, which consequently have a predisposition to develop a Brachycephalic Obstructive Airway Syndrome (BOAS) (7–10). It is remarkable that this disease is one of the most common causes of shortened life expectancy in these breeds (11, 12). In a study on the quality of life of brachycephalic dogs, the owners reported in particular reduced physical activity, sleep disturbances and sensitivity to heat in their animals (13). An increased risk of breathing disorders during sleep is also reported (14). In fact, brachycephalic dogs have already been used as a natural model for sleep apnoea syndrome (OSA) in humans (15, 16).

The increase in intrathoracic pressure due to upper airway obstruction may also cause gastrointestinal symptoms such as ptyalism, regurgitation and vomiting (17, 18). Diagnosis is based on symptoms, clinical examination and diagnostic imaging (19). The treatment of choice is surgical correction of anatomical defects, such as widening the nostrils or shortening the soft palate (20).

BOAS is widespread and associated with pain, suffering and harm to the animals (21). To prevent breeding with diseased dogs, it is necessary to identify early signs of airway obstruction, which are not always apparent under resting conditions (16). In fact, several studies have demonstrated the utility of a submaximal exercise test to more accurately diagnose dogs with BOAS (16, 21–23). Identifying the intensity and nature of breath sounds to accurately assess the degree of BOAS during exercise testing is based on a careful clinical examination that requires a great deal of experience (21).

A number of parameters can be used to assess physical stress in the context of physical exercise, as has already been used, for example, in studies on sled dogs (24, 25). Cortisol is a well-known stress hormone, that is controlled by the hypothalamic–pituitary–adrenal (HPA) axis (26). It has already been used in both human and veterinary medicine to assess acute physical and psychological stress and pain (10, 27–29). Fluctuations in cortisol levels can be tracked by the concentrations in serum and saliva and correlate with each other (30, 31). Cortisol levels in dogs can be influenced by different physiological or environmental factors, e.g., an unfamiliar environment or the absence of the owner (27).

BOAS is often not recognized by the owner and for this reason often not diagnosed (32, 33). To the authors’ knowledge, cortisol measurements related to stress in brachycephalic dogs have not been studied in the context of physical exercise. The aims of the study were therefore to evaluate the procedure of a standardized stress test and to detect BOAS predisposed dogs at an early stage. Cortisol concentrations in the saliva of the dogs were also examined to obtain indications of acute as well as chronic stress in the dogs.

## 2 Materials and methods

All studies and treatments were approved by the Ethics Committee of the Centre for Clinical Veterinary Medicine, Faculty of Veterinary Medicine, Ludwig-Maximilians-University Munich (application 167-02-05-2019).

### 2.1 Study protocol

The study was conducted from May 2019 to November 2021 at the Surgical and Gynaecological Small Animal Clinic, Ludwig-Maximilians-University Munich, Germany. Inclusion criteria for the study group were healthy purebred Pugs and French Bulldogs older than 2 years, with no previous diseases or surgical treatment of the respiratory tract. Small, dolicho or mesocephalic dog breeds that were lighter than 15 kg and older than 2 years served as control group, also without previous diseases.

**Figure 1:**
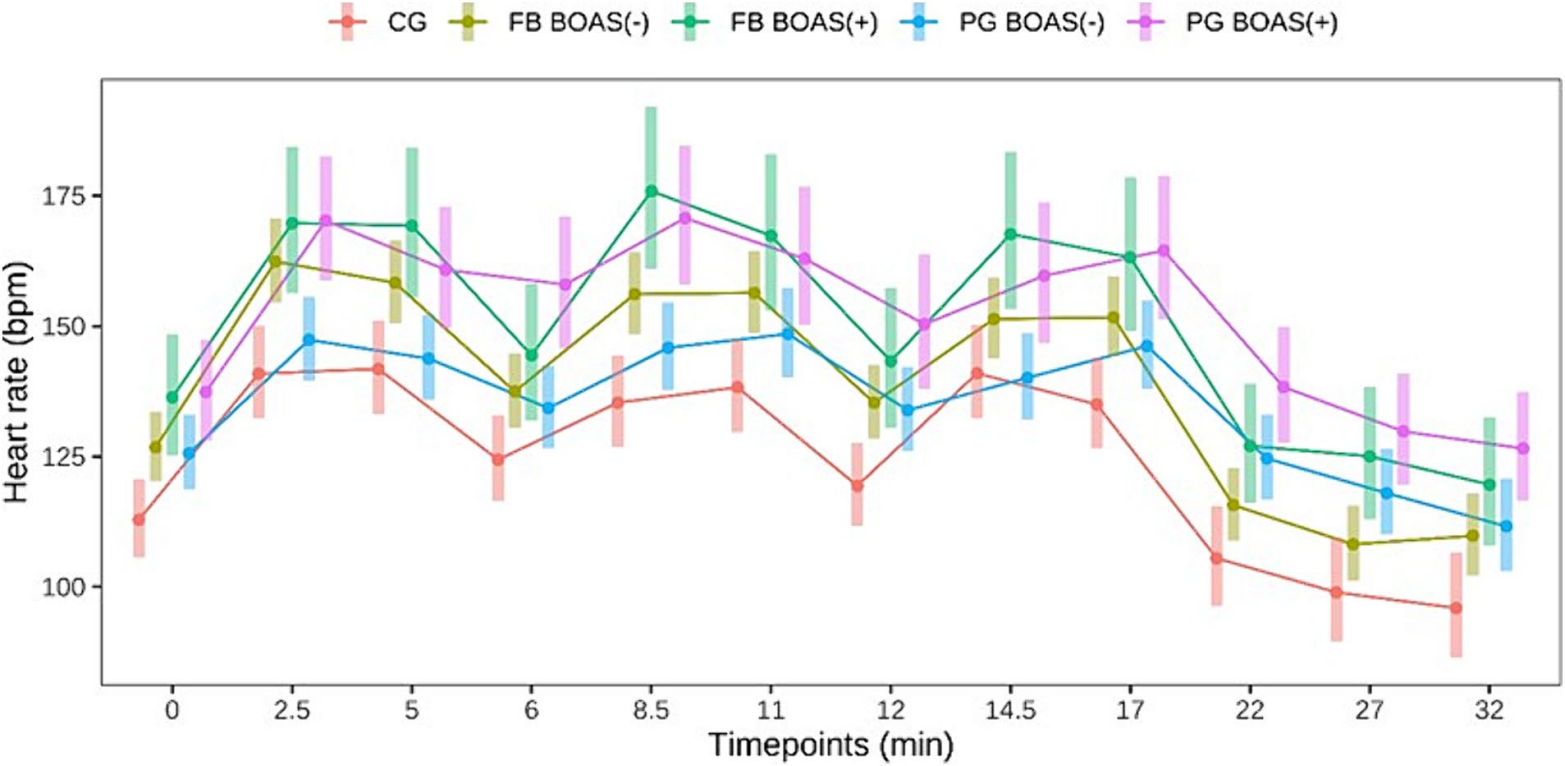
Comparison between the heart rate (bpm) of PG, FB and CG at rest, during and after the exercise test. The exercise takes place from minute 0 to 17.

As part of the study, a medical history questionnaire was completed, a clinical examination and a standardized stress test were performed (16) and saliva samples were collected at specific time points (Figure 1).

The questionnaire consisted of an assessment of the owner’s perception of breathing sounds, limitations in physical fitness and concomitant diseases. Owners were asked about the dog’s breathing sounds when awake and asleep, the subjective degree of limited physical activity and the perceived level of stress.

A general examination including respiratory and heart rate, throat and thoracic auscultation and measurement of body temperature was performed as part of the preliminary examination. A heart rate belt (Polar H10 Electro, Kempele, Finland) was placed on the patient’s chest to measure the pulse rate continuously. Body temperature was measured with a digital thermometer. The weight of all dogs was recorded and the body condition score (BCS) was determined on a scale of 1 to 9 points (34). A modified stress test was used to assess the degree of BOAS (16), based on the method originally developed and validated by Liu et al. (35). In the 10-min adaptation phase, the dogs first got used to the environment and learned to run on the treadmill. This was followed by a 15-min break before the stress test was performed (16).

In the first step of the study, both the stress test results and the cortisol measurements of the Pugs and French Bulldogs were compared with the animals of the control group. In the second step, an internal comparison was made between the two brachycephalic breeds, divided into BOAS(+) (suffering from BOAS) and BOAS(−) (not suffering from BOAS).

### 2.2 Standardized stress test

A ventilated room with a constant temperature between 20 and 24°C was chosen for the test. The exercise test was performed using a motorized treadmill (Kistler Instrumente GmbH, Munich, Germany). It lasted a total of 17 min, taking into account the 15 min of running and the two breaks of 1 min each after 5 and 10 min of running time. During the exercise test, the dogs trotted at their individual minimum speed on the treadmill (16). The owner of the dog was present during the entire test to avoid an additional stress factor. Dogs that refused to run on the treadmill were excluded from the test.

Heart rate was measured with the help of a pulse belt throughout the test. The heart rate should increase by at least 40% of the initial value during the stress test to ensure a minimum workload (36). The exercise test was not performed or was terminated if the dog was tachycardic with a sustained heart rate above 220 beats per minute, dyspnoeic with difficult breathing or showed continuous stertor or stridor before or during the test.

After the exercise test (minutes 0–17), a 15-min rest period was established from minute 18 to minute 32, during which heart rate and respiratory rate were measured every 5 min in all dogs. For subjects that did not show a return to basal levels of these parameters, measurements were taken beyond minute 32 until normalization with a 10% tolerance (16).

### 2.3 Cortisol measurements

All dogs had the opportunity to spend a 15-min adaptation period in the treadmill room with the owner, at the end of which the first saliva sample was taken. Saliva samples were taken immediately before and after the test (minute 17) and 15, 30 and 60 min after the test, i.e., at minutes 32, 47 and 77 using commercially available swabs (Salimetrics Children’s Swab and Tubes, Pennsylvania, United States). The swabs were left in the animal’s mouth for about 1 min, then transferred to appropriate tubes, centrifuged at 500 rpm, and saliva was then stored in an Eppendorf tube at −20 degrees until analysis. The saliva samples were analyzed in the in-house laboratory using an antibody-based ELISA kit (Cortisol Enzyme Immunoassay Kit, Salimetrics, Pennsylvania, United States) according to the manufacturer’s instructions (37). For this purpose, the ELISA plate was first washed twice, the samples diluted with an appropriate buffer solution and then pipetted onto the ELISA plate. Afterwards, the Biotin-detection-Antibody (BDA) working solution was pipetted and the plate incubated covered at 37°C for 45 min. In the next step, the ELISA plate was washed 3 times and then the Streptavidin-horseradish peroxidase conjugate solution was pipetted and the plate was incubated again at 37°C for 30 min. After washing the plate 5 times, the tetramethylbenzidine substrate, which was also pre-warmed at 37°C, was pipetted and the plate was covered again and incubated at 37°C for 15 min. Finally, a stop solution was pipetted and the plate was immediately measured (ELISA Reader Infinite® F50, Tecan Deutschland GmbH, Crailsheim, Germany).

## 3 Statistical analyses

Data analysis was performed using R 3.6.3 (2020-02-29). Results with a *p*-value <0.05 were considered statistically significant, while with *p*-values between 0.05 and 0.1 were considered a tendency. We used generalized linear mixed-effects models with negative binomial distributions family to predict counts of heart and breeding frequencies over time. Negative binomial family was chosen over poisson due to the violation of overdispersion assumption (38). Temperature was modeled via generalized linear mixed-effects models with Gaussian distribution family, due to no violations in assumptions: approximately normal distribution of the residuals and no heteroscedasticity (39). We used generalized linear models with Gamma family (due to continuous and highly skewed distribution) to predict cortisol concentrations. Mixed models included individual animals as random effect fitted on the intercept. *p*-values after comparing pairwise categories were always adjusted with Tukey method for multiple comparisons.

## 4 Results

140 dogs were examined in this study. A total of 57 Pug dogs (PG) and 56 French Bulldogs (FB) were included. As a control group (CG), 27 non-brachycephalic dogs of similar age and body weight to the study population were included in the study.

Among the 57 PG, 25 males, six of which were neutered, and 32 females, 11 of which were neutered, the mean age was 6 years (range 2–14), the mean weight was 8 kg (range 4–12) and the mean BCS was 5/9 (range 4–7/9).

Regarding the medical history, none of the PG owners reported that their PG’s quality of life was affected by serious illness or previous surgery, although 17/57 (10%) owners reported moderate to severe breathing problems at rest and 22/57 (13%) during sleep. 4/57 (7%) noted moderate to severe exercise intolerance in their dog, while 12/57 (21%) experienced high levels of stress and agitation.

Among the 56 FB, 25 males, ten of whom were neutered, and 31 females, nine of whom were neutered, the mean age was 4 years (range 2–10), the mean weight was 12 kg (range 7–19) and the mean BCS was 5/9 (range 4–7/9). Based on the medical history, 12/56 (21%) FB had moderate to severe respiratory symptoms at rest and 17/56 (30%) at sleep. Moderate to severe exercise intolerance due to physical fitness was felt by 9/56 (16%) of the owners, while 15/56 (14%) of them reported high levels of stress and restlessness.

The CG, 8 males of which 5 were neutered and 19 females of which 6 were neutered, included eight Parsons Russell Terriers, four Jack Russell Terriers, four mixed breed dogs, three Beagles, three Dachshunds and one each of Bolonka, Coton de Tuléar, Poodle and West Highland White Terrier. The average age was 6 years (range 2–15 years), the average weight was 9 kg (range 5–20) and the average BCS was 5/9 (range 4–8/9). None of the owners reported that their dog’s quality of life was affected by respiratory problems, exercise intolerance or high levels of stress.

### 4.1 Severity of BOAS

The dogs were classified into BOAS classes. in the P group 14/57 (25%) were classified as “without signs” of BOAS, 21/57 (37%) as “with mild” signs, 18/57 (32%) as “with moderate” signs and 4/57 (7%) as “with severe” signs of BOAS. In the FB group, 19/56 (34%) dogs were classified as “with no signs” of BOAS, 22/56 (39%) as “with mild” signs, 13/56 (23%) as “with moderate” signs and 2/56 (4%) as “with severe” signs of BOAS. Dogs with no or mild signs of BOAS were classified in this study as “BOAS(−),” i.e., not clinically significant, dogs with moderate or severe signs of BOAS were classified in this study as “BOAS(+),” i.e., clinically affected (7). Thus, 35 out of 57 (61%) PG or 41 out of 56 (73%) FB fell into the BOAS(−) group and 22 out of 57 (39%) P or 15 out of 56 (27%) FB fell into the BOAS(+) group. All dogs in the control group were classified as BOAS(−).

### 4.2 Fitness test

16 out of 57 PG (28%) could not complete the exercise test: nine were excluded due to a heart rate above 240 bpm, one developed extreme respiratory distress and loud wheezing, the remaining six dogs were unable to complete the exercise test due to high stress. Ten of these 16 dogs were BOAS(+). Six of 56 French Bulldogs (11%) failed the stress test, two had severe respiratory distress, one had a persistently high heart rate of >220, and three dogs had to stop the test due to severe exhaustion. One of the control dogs (4%) failed the test due to a persistently high heart rate.

41 out of 57 PG could complete the test, 29 of them were BOAS(−) and 12 BOAS(+). Of the remaining 50 FB that completed the test, 39 were BOAS(−) and 11 were BOAS(+). In all dogs that completed the run, the heart rate had returned to baseline after 15 min of rest, with a tolerance of 10%. Three dogs one PG BOAS(−), one PG BOAS(+) and one FB BOAS(−) showed a higher respiratory rate even without loud breathing sounds, up to 30 min after the run.

#### 4.2.1 Comparison between BOAS (+) and non BOAS (−) brachycephalic dogs and non-brachycephalic dogs

##### 4.2.1.1 Heart rate, respiratory rate and body temperature

Heart rate before, during and after the test was significantly higher in BOAS(+) PG than in BOAS(−) (*p* < 0.05). With respect to FBs, heart rate was significantly higher in BOAS(+) than in BOAS(−) at the times of greatest physical exertion (*p* < 0.05), while it tended to converge between the two groups after breaks, especially at minute 6 (*p* = 0.19) and 12 (*p* = 0.15). The heart rate curves of both races are shown in Figure 1.

**Figure 2:**
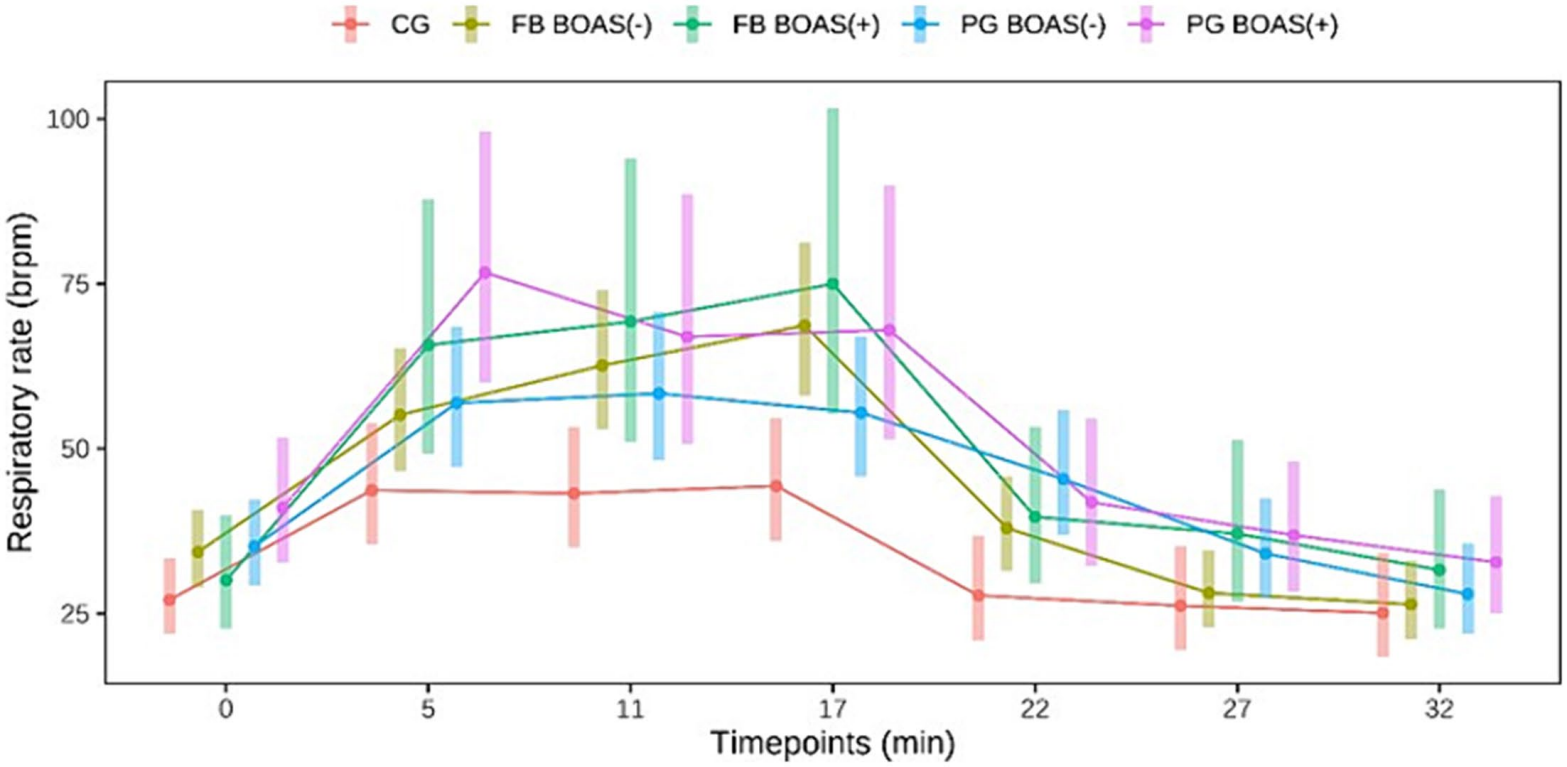
Comparison between the respiratory rate (brpm) of PG, FB and CG at rest, during and after the exercise test. The exercise takes place from minute 0 to 17.

Regarding the respiratory rate in PG, the BOAS(+) showed a higher respiratory rate than the BOAS(−) during the test, while a higher respiratory rate was observed in the BOAS(−) PG after the test. However, the results were not statistically significant (*p* > 0.06). Also in FB, no statistically significant difference in respiratory rate was observed in the BOAS(+) and BOAS(−) dogs. The latter had a lower frequency throughout the test and at rest. The curve of the respiratory rate of the two breeds is shown in Figure 2.

**Figure 3:**
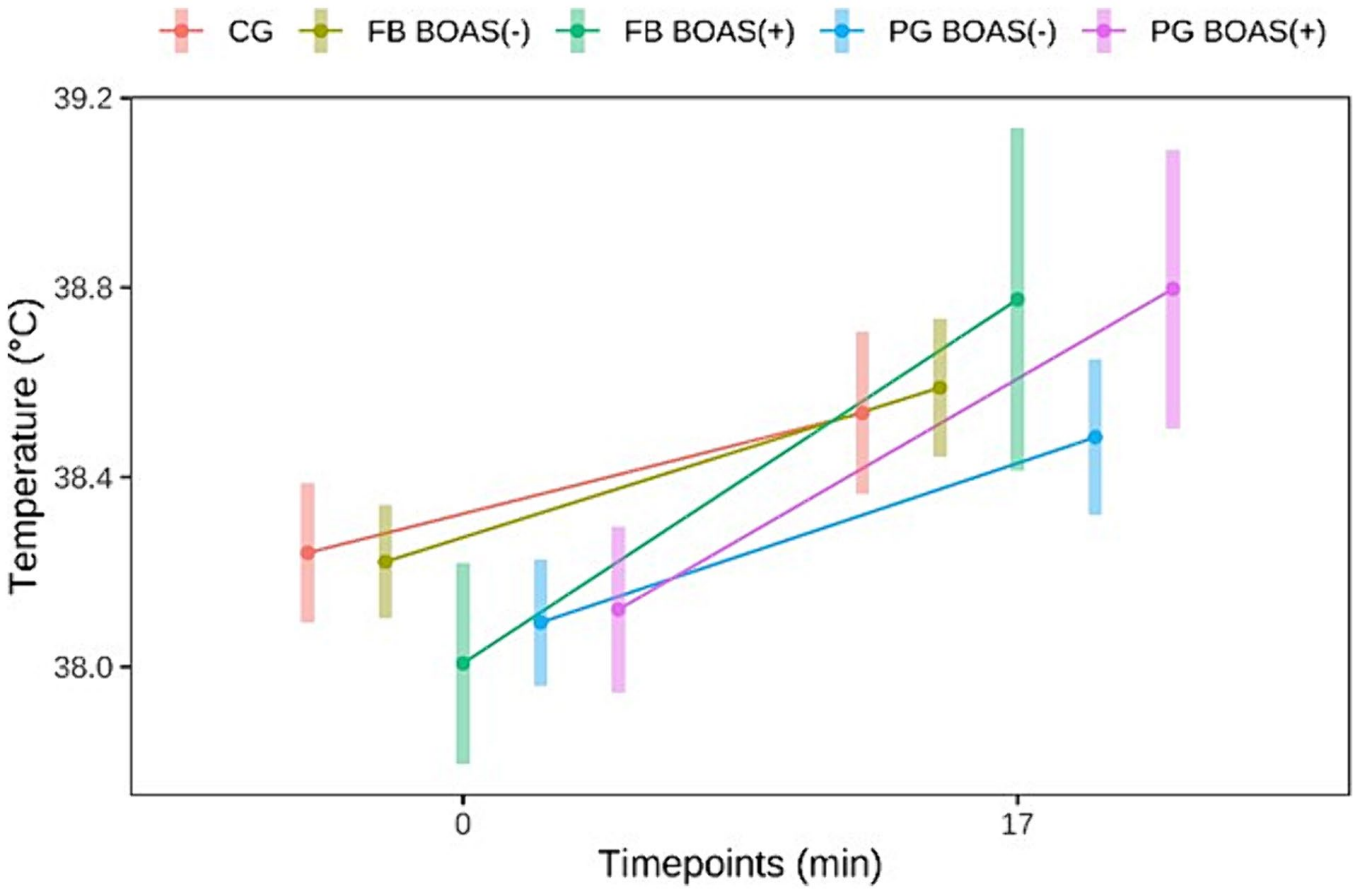
Comparison between the temperature (°C) of PG, FB and CG at rest and after the exercise test.

The body temperature before and after exercise is shown in Figure 3. The difference in the change of body temperature, before and after exercise, was statistically significant in PG and FB (*p* < =0.002).

##### 4.2.1.2 Salivary cortisol measurements

**Figure 4:**
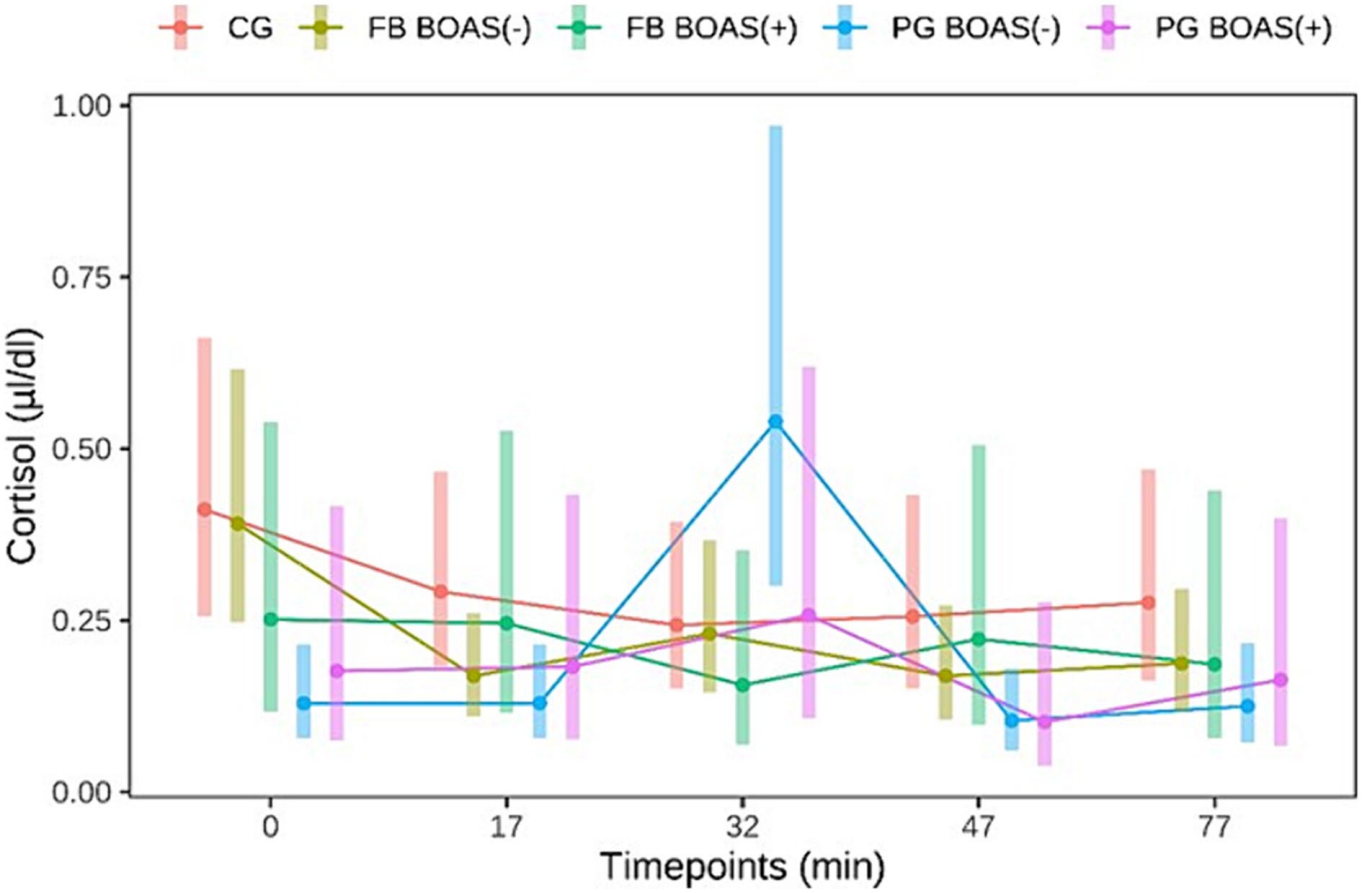
Comparison between the cortisol values (μl/dl) of PG, FB and CG before and after the exercise test. The exercise takes place from minute 0 to 17.

The measurement of cortisol was performed on a total sample of 87 dogs. Of these 87 dogs 27 were PG, 36 FB and 24 CG. Dogs that did not complete the exercise test and all those whose saliva samples were not sufficient for measurement with the antibody test were excluded from this measurement. Salivary cortisol levels in PG, as shown in Figure 4, showed a significant difference between BOAS(+) and BOAS(−) immediately before the test (minute 0), immediately after exercise (minute 17) and after 1 h of rest (*p* < 0.05). The cortisol concentrations of the PG with BOAS(−) rose significantly during the test and then fell again. In contrast, the cortisol concentrations of the PG with BOAS(+) were higher before and after the test, but did not show a statistically significant increase or decrease (*p* > 0.8).

With respect to FB, the BOAS(−) group showed significantly higher salivary cortisol levels before the test than the BOAS(+) group (*p* = 0.037). During the test, the dogs in the BOAS(−) group showed a significant decrease in cortisol compared to time 0 (*p* = 0.002). The BOAS(+) dogs showed no significant change in salivary cortisol.

##### 4.2.1.3 Probability of failing the exercise test

**Figure 5:**
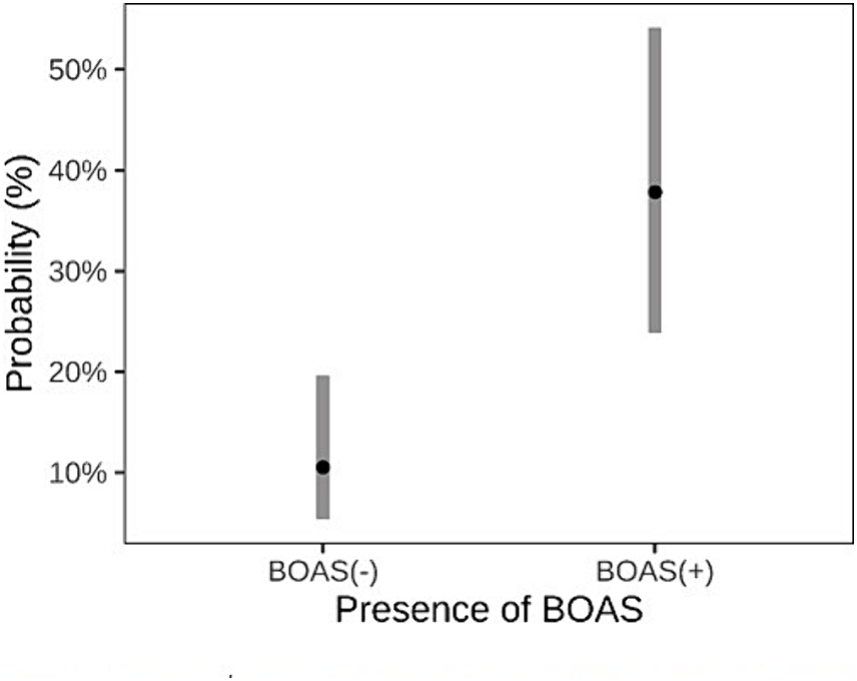
Comparison between dogs classified as BOAS(+) and BOAS(−) regarding the predicted probability of failing the exercise test.

Brachycephalic dogs BOAS(+) had a higher rate of failure in the exercise test (14/27) compared to those BOAS(−) (8/76), resulting in an odds ratio of 5.2 (95% CI, 1.97–14.50, *p* = 0.001). The probability for a BOAS (−) dog to fail the exercise test was 10.5%, while the probability for a BOAS (+) dog to fail the exercise test was 37.8% as represented in Figure 5.

## 5 Discussion

The prevalence of brachycephalic breeds has increased greatly in recent decades, accompanied by an increase in the characteristics of brachycephaly (21, 40, 41). Breeding dogs with pronounced physical features of brachycephaly and BOAS has inevitably led to a deterioration in the quality of life (8, 9). It is therefore necessary to develop testing options to distinguish healthy individuals from those that are not suitable for breeding at an early stage. Several authors have already investigated a non-invasive predictive test that can be used to distinguish between BOAS-affected and non-affected dogs (16, 21, 35, 42). In a previous study (42) barometric whole-body plethysmography was used as a non-invasive and objective technique. Subsequently, the same group of researchers validated the stress test and laryngeal auscultation as an objective measurement method to determine the BOAS severity level (21). The authors advocated the use of the 3-min trot test, a low-cost test that increases the sensitivity of the clinical examination to determine BOAS severity. This allows affected dogs to be identified and treated more quickly and the effectiveness of surgery to be more accurately assessed (21). However, the clinical assessment of exercise intolerance, breath sounds and recovery time is still not completely objective, but depends largely on the experience of the examiner (21). In the present study, however, a correlation between the inability to complete the exercise test and the dogs’ probability of having BOAS was found, confirming the efficacy of a stress test in differentiating affected from unaffected dogs. The use of technologies such as whole-body pletysmography are particularly accurate and reliable, but also more complex and expensive (21). Hence, there is still a need for a standardized examination procedure for the diagnosis of brachycephaly that is reliable, repeatable and affordable for the dog owner everywhere (16).

To the authors’ knowledge, salivary cortisol has not yet been studied in relation to exercise in brachycephalic breeds. The aim of the present study was therefore to find out whether vital signs and salivary cortisol concentrations could be used in a standardized, non-invasive exercise test to distinguish between healthy dogs and those suffering from BOAS.

### 5.1 Heart rate, respiratory rate, and body temperature

During exercise, CG showed a significantly lower heart rate than BOAS(+) brachycephalics and FBs, while during the recovery phase the heart rate of PGs remained significantly higher than that of CG, indicating that FB recover faster. Explanations for a comparatively poor physical condition also emerge from previous studies on PG. Compared to other brachycephalic breeds, PG have a particularly strong predisposition to develop laryngeal collapse and bronchial wall thickening (BWT) (43–45). In addition, a positive correlation between BOAS and BWT was found in a previous study (44). Two recent British studies (4, 46) found that PG have a greater predisposition to developing BOAS than FBs. Thus, the two studies found that PG have about a 54-fold higher risk of developing BOAS than non-PG, while FB have about a 31-fold higher risk than non-FB.

As for the BOAS(+) dogs, both studied breeds showed a higher heart rate than the BOAS(−) dogs for almost the entire duration of the test. This result correlates with that of a previous study (16) and may indicate that the body condition of the brachycephalic dogs is impaired when they show mild to severe symptoms of BOAS.

The CG had a significantly lower heart rate at most time points of the test, indicating a lower oxygen demand of the organism under stress. In terms of respiratory rate, brachycephalic breeds showed a significantly higher rate than the control group. It can be assumed that, as already examined in previous studies, dogs of non-brachycephalic breeds show better physical fitness on average (47) or are healthier (48). Also previous studies reported that brachycephalic dogs with upper airway obstruction have lower exercise capacity than dolicho- and mesocephalic dogs (49–52).

Heart rate variability (HRV) is a parameter that has been used in previous studies to assess the stress response in dogs, however, the utility of this parameter in dogs during exercise has yet to be determined. In a recent study, HRV in brachycephalic breeds was investigated, and according to the authors the dog’s breed and morphology did not alter the HRV (53). Future studies are needed to determine the usefulness of HRV and its correlation with salivary cortisol in brachycephalic dogs undergoing a fitness test (54, 55). The evaluation of breath sounds should only be carried out by trained personnel, to avoid dogs with BOAS passing the test (21). In the present study, the assessment of breath sounds and wheezing was also carried out through the subjective perception of the trained examiners. All examinations were performed by the same two examiners, trained in the assessment of BOAS dogs during the same time period, according to the standards of the same university facility.

### 5.2 Cortisol

Cortisol in saliva has already been investigated in other studies assessing stress in dogs (25, 27, 56–60). For example, one study examined serum cortisol in relation to physical exertion in sled dogs (25). The results showed a significant increase in cortisol after exertion. A less invasive sampling was chosen in the present study, as multiple blood sampling to measure serum cortisol would have represented an additional stress factor. Salivary cortisol, as an alternative to serum cortisol, has been investigated in previous studies as a marker to assess stress in dogs (27, 56–60). In the study by Csoltova et al. (59) the cortisol levels in the saliva of dogs were measured before and after the veterinary examination at an interval of about 12–13 min. The authors found no significant difference between the two measurements, which was probably due to a too short time interval or the low intensity of the stimulus, which may not have been sufficiently stressful. In another Canadian study, plasma and salivary cortisol were compared in a group of dogs, before and immediately after air transport (56). In this study, it was found that plasma and salivary cortisol levels increased significantly after transport in both awake and sedated dogs, which they attributed to the high intensity of the stimulus. With regard to separation anxiety, a study by Shin and Shin in 2016 observed that dogs separated from their owners had significantly higher salivary cortisol peaks, after 20 min, than dogs that could smell and hear their owners (60). Therefore, in the present study, the owner was present for the entire duration of the test, in order to avoid an additional stress factor for the dogs examined.

The present study shows that different breeds exhibited different cortisol curves in the context of a standardized stress test. Considering only the non-BOAS affected animals, the PG showed a significant cortisol increase about 15 min after the end of the exercise, while the bulldogs showed a significant decrease in cortisol 15 min after running. Concerning the increase in cortisol levels, considering the cortisol values obtained from the start of physical activity, it can be seen in Figure 4 that PG BOAS(−) reach cortisol peaks, following physical activity, above 0.5 μL/dL, a value never reached by the other two groups of dogs. It might be speculated that the stimulus of the test might be sufficient for PG, which among the two brachycephalic breeds are considered the less healthy ones (4, 46), but not for FB. For some dogs, an unfamiliar environment can itself be a stressor (61). However, in the present study, a significant reduction in cortisol values from baseline was observed only in the FB BOAS group (−). This result can be interpreted with increased initial stress despite the presence of the owner, and/or insufficient intensity of exercise. Interestingly, the BOAS(+) dogs showed no significant change in cortisol during the test compared to the BOAS(−) dogs. This result could indicate a decreased responsiveness of the HPA axis in dogs with BOAS since previous studies have shown that, after prolonged HPA stimulation, such as occurs with chronic pain and stress, cortisol release decreases in both humans and animals (62–65). Further studies, comparing measurements of different markers of acute and chronic stress, are needed to confirm this hypothesis.

Cortisol secretion is regulated by negative feedback from the paraventricular hypothalamic nucleus via corticotropin-releasing hormone (CRH), adrenocorticotropic hormone (ACTH) and the secretory cells of the adrenal cortex (66). Sustained high levels of cortisol have an inhibitory effect on the central and pituitary levels of the HPA axis, so the pituitary can become increasingly resistant to CRH and ACTH (67–69). As reported in previous studies, brachycephalic dogs represent a human model of sleep apnoea syndrome (OSAS), in which patients experience a state of hypoxia at night due to respiratory distress (15). Hypoxia in OSAS, such as in Chronic Fatigue Syndrome (CFS) (70, 71) is a state of stress that, if prolonged, can alter HPA axis function and circadian cortisol rhythms (72–74). The present study aimed to measure acute stress, assumed that the major stress factor for brachycephalic dogs is respiratory distress and that this may be exacerbated by an exercise test. Regarding chronic stress, on the other hand, and its influence on the results obtained, no information is available. However, taking the four groups of brachycephalic dogs into consideration, only those considered healthy show significant changes in cortisol values. It could be speculated that the dogs most affected by airway obstruction might exhibit altered activity of the HPA axis. Further investigations to assess the HPA axis were not carried out in the present study, which is why no statements can be made on this.

Cortisol levels can be influenced by a variety of factors, including the circadian rhythm of cortisol and individual variability. Previous studies have shown that cortisol in dogs peaks in the morning, decreases throughout the day, and reaches its minimum in the evening (75, 76). However, some other studies have described the lack of circadian and diurnal variations in cortisol levels (56, 77–79). No conclusion can be drawn about this in the present study, since the investigation determined the time of the test, based on the availability of the owners. In general, all examinations took place in the late morning or early afternoon, i.e., neither early in the morning nor late in the evening.

Different temperament, age, breed, personality and individual previous experiences also influence the extent of the perceived stress in the dog and thus its expression (80, 81). According to Cobb et al. (27) when assessing cortisol levels in dogs, individual changes in cortisol levels should be interpreted with caution. The investigations in the present study were carried out in an unfamiliar environment, namely in a veterinary clinic. In order to reduce the dogs’ stress as much as possible, the dogs did not have to wait in a waiting room, but instead entered the gait laboratory directly via a side entrance. The owners were permanently present during the test and the dogs were initially accustomed to the treadmill with a lot of time and patience. Also, only dogs that well tolerated the trot on the treadmill were included. Ideally, follow-up studies should be conducted by trained personnel in a familiar environment to reduce additional stressors. Therefore, future studies might consider performing multiple tests on the same dog at regular intervals, to compare possible varying results. The owner participation in the current study was voluntary, a larger number of subjects from each BOAS group could be included in follow-up studies.

To determine each subject’s basal cortisol, multiple measurements should be taken over a 24-h period while the dog is in its familiar environment. In order to standardize the test for further studies, owners should be trained and have at least 24 h availability for complete sample collection.

In addition to cortisol, many other parameters can be used to assess a stressful situation. Within the sympathetic-adrenal system (SAM), the sympathetic and parasympathetic nervous systems are activated first (82). The release of catecholamines (epinephrine and norepinephrine) and the activation of the sympathetic nervous system serve to regulate blood pressure by contracting the smooth muscles in the blood vessels. Catecholamines cause increased contractility of the heart muscle and modulate the metabolism to increase blood sugar levels by stimulating glycogenolysis in the liver, increasing glucagon secretion, decreasing insulin secretion from the pancreas and stimulating lipolysis in adipose tissue (83). These physiological changes are associated with “fight-flight” responses in dogs and humans (84, 85). Chromogranin A (CgA) is released together with catecholamines during acute stress. Therefore, the CgA epitopes, catestatin (CST) and vasostatin (VS) could be measured in dog saliva to assess a stressful situation (88). It should be noted that sufficient sample material must be available to measure the relevant parameters. In the present study, only one parameter could be measured due to the small amount of saliva collected.

Besides, it is not to forget that taking a saliva sample can also be associated with stress for the animal (86, 87). For this reason, it was possible to measure cortisol only in dogs that tolerated saliva sampling for the required time. Further studies and development of new materials, dedicated to veterinary medicine, are necessary in order to optimize saliva collection in dogs.

In conclusion, the assessment of vital signs during a standardized fitness test can be useful to differentiate between dogs suffering from BOAS and healthy brachycephalic dogs. However, affected dogs can also manage the 15-min trot and signs of BOAS, such as labored breathing and breath sounds should therefore be evaluated by experienced examiners. Whether and how the functionality of the HPA axis is limited in chronically ill BOAS patients, needs to be investigated in subsequent studies with long follow-up periods.

## Data availability statement

The raw data supporting the conclusions of this article will be made available by the authors, without undue reservation.

## Ethics statement

The animal studies were approved by the Ethics Committee of the Centre for Clinical Veterinary Medicine, Faculty of Veterinary Medicine, Ludwig-Maximilians-University Munich. The studies were conducted in accordance with the local legislation and institutional requirements. Written informed consent was obtained from the owners for the participation of their animals in this study.

## Author contributions

EK: Data curation, Investigation, Resources, Writing – original draft. AM-L: Conceptualization, Funding acquisition, Supervision, Writing – review & editing. YZ: Formal analysis, Writing – review & editing. MS: Investigation, Project administration, Resources, Writing – review & editing.
